# H3K9ac of TGFβRI in human umbilical cord: a potential biomarker for evaluating cartilage differentiation and susceptibility to osteoarthritis via a two-step strategy

**DOI:** 10.1186/s13287-021-02234-8

**Published:** 2021-03-04

**Authors:** Yongjian Qi, Bin Li, Yinxian Wen, Xu Yang, Biao Chen, Zheng He, Zhe Zhao, Jacques Magdalou, Hui Wang, Liaobin Chen

**Affiliations:** 1 Department of Orthopedic Surgery, Zhongnan Hospital of Wuhan University, Wuhan, 430071 China; 2 Hubei Provincial Key Laboratory of Developmentally Originated Disease, Wuhan, 430071 China; 3 UMR 7561 CNRS-Université de Lorraine, Faculté de Médicine, Vandoeuvre-lès-Nancy, France; 4 Department of Pharmacology, Wuhan University School of Basic Medical Sciences, Wuhan, 430071 China

**Keywords:** Biomarker, Chondrogenic differentiation, Osteoarthritis, Transforming growth factor β receptor I, Wharton’s jelly-derived mesenchymal stem cells

## Abstract

**Background:**

Epidemiological investigation and our previous reports indicated that osteoarthritis had a fetal origin and was closely associated with intrauterine growth retardation (IUGR). Human Wharton’s jelly-derived mesenchymal stem cells (WJ-MSCs) could be programmable to “remember” early-life stimuli. Here, we aimed to explore an early-warning biomarker of fetal-originated adult osteoarthritis in the WJ-MSCs.

**Methods:**

Firstly, two kinds of WJ-MSCs were applied to evaluate their chondrogenic potential in vitro through inducing chondrogenic differentiation as the first step of our strategy, one from newborns with IUGR and the other from normal newborns but treated with excessive cortisol during differentiation to simulate the excessive maternal glucocorticoid in the IUGR newborns. As for the second step of the strategy, the differentiated WJ-MSCs were treated with interleukin 1β (IL-1β) to mimic the susceptibility to osteoarthritis. Then, the expression and histone acetylation levels of transforming growth factor β (TGFβ) signaling pathway and the expression of histone deacetylases (HDACs) were quantified, with or without cortisol receptor inhibitor RU486, or HDAC4 inhibitor LMK235. Secondly, the histone acetylation and expression levels of TGFβRI were further detected in rat cartilage and human umbilical cord from IUGR individuals.

**Results:**

Glycosaminoglycan content and the expression levels of chondrogenic genes were decreased in the WJ-MSCs from IUGR, and the expression levels of chondrogenic genes were further reduced after IL-1β treatment, while the expression levels of catabolic factors were increased. Then, serum cortisol level from IUGR individuals was found increased, and similar changes were observed in normal WJ-MSCs treated with excessive cortisol. Moreover, the decreased histone 3 lysine 9 acetylation (H3K9ac) level of TGFβRI and its expression were observed in IUGR-derived WJ-MSCs and normal WJ-MSCs treated with excessive cortisol, which could be abolished by RU486 and LMK235. At last, the decreased H3K9ac level of TGFβRI and its expression were further confirmed in the cartilage of IUGR rat offspring and human umbilical cords from IUGR newborn.

**Conclusions:**

WJ-MSCs from IUGR individuals displayed a poor capacity of chondrogenic differentiation and an increased susceptibility to osteoarthritis-like phenotype, which was attributed to the decreased H3K9ac level of TGFβRI and its expression induced by high cortisol through GR/HDAC4. The H3K9ac of *TGFβRI* in human umbilical cord could be a potential early-warning biomarker for predicting neonatal cartilage dysplasia and osteoarthritis susceptibility.

**Supplementary Information:**

The online version contains supplementary material available at 10.1186/s13287-021-02234-8.

## Background

Osteoarthritis is a chronic joint disease characterized by articular cartilage degeneration, which has long been considered as an age-related degenerative disease. However, epidemiological investigations have shown that osteoarthritis of multiple joints, such as the hand and hip, is closely associated with low birthweight [1–5]. Intrauterine growth retardation (IUGR) refers to fetal growth restriction caused by various prenatal adverse factors, with the main manifestations being multiple organ developmental dysfunction, growth retardation, and low birthweight [6]. IUGR diagnosis criterion is that baby weight at 10% or two or more standard deviations less than the mean body weight of normal babies at the same gestational age [7, 8]. Our previous studies found that prenatal exposure to xenobiotics (e.g., caffeine, nicotine, and ethanol) and food restriction could result in IUGR of rat offspring [9–14], and the IUGR rats exhibited persistent cartilage dysplasia and increased susceptibility to osteoarthritis in adulthood [14–22]. All these reports indicate that osteoarthritis has a fetal origin [23]. Considering the substantial healthcare resources and costs associated with osteoarthritis therapy [24], it is necessary to explore the early-warning marker of fetal-originated osteoarthritis, which could change the current strategy for osteoarthritis prevention by targeting early-life factors.

It is known that prenatal baseline levels of glucocorticoids (cortisol in humans and corticosterone in rodents) play an important role in the morphological and functional maturation of fetal tissues [25]. However, high levels of serum glucocorticoids could cause abnormal fetal development [25]. Several studies have demonstrated that increased level of glucocorticoids is positively correlated with the incidence of IUGR in fetuses [26, 27]. “Intrauterine programming” refers to the long-term or permanent functional changes in an individual due to adverse prenatal conditions during fetal development [27]. Multiple reports suggested that excessive maternal glucocorticoid might be the trigger for intrauterine programming and that excessive glucocorticoids could induce long-term changes in the expression of multiple genes through genetic imprinting, which leads to persistent changes in fetal structure and function [28, 29]. Furthermore, it has been indicated that epigenetic alterations might act as more stable and reliable molecular markers of early-life events than the expression of the target genes [30]. Our previous studies have confirmed the “excessive maternal glucocorticoid” phenomenon in IUGR offspring with prenatal xenobiotic exposure, which might trigger the susceptibility to osteoarthritis of these IUGR offspring [13, 31, 32]. Accordingly, we speculated that fetal-originated osteoarthritis might be attributed to the alterations in epigenetic programming induced by maternal glucocorticoid overexposure. The abnormal epigenetic alterations could be early biomarkers for predicting the adult osteoarthritis with fetal origination.

Human Wharton’s jelly-derived mesenchymal stem cells (WJ-MSCs) are multipotent and can be induced to differentiate into chondrocytes in vitro [33, 34]. Increasing evidence have indicated that stem cells, including WJ-MSCs, could be the targets of inappropriate environments and might be programmable to “remember” early-life stimuli that would affect their function in adult life [35–38]. Moreover, several reports have suggested that human WJ-MSCs from small for gestational age infants might preserve some identifiable molecular pathways and epigenetic markers [37, 39]. These results indicated that human WJ-MSCs might be appropriate for developing a cell model in vitro, to elucidate potential molecular mechanisms of the fetal origination of adult osteoarthritis and predict cartilage dysplasia and subsequent susceptibility to adult osteoarthritis.

In this study, we established a two-step model based on three-dimensional chondrogenic differentiation of WJ-MSCs to mimic cartilage development in utero and the inflammatory stimulation that had occurred under unfavorable conditions in adulthood in vivo. We aimed to investigate the capacity of chondrogenic differentiation of human WJ-MSCs from IUGR newborns and the subsequent susceptibility to an osteoarthritis-like phenotype. Furthermore, we sought to elucidate the initial factor and potential pathway programmed by epigenetic modification changes involved in these phenomena. Finally, the epigenetic imprinting was verified in the rat IUGR models and human umbilical cord with IUGR, which provided a promising early-warning biomarker for fetal-originated adult osteoarthritis.

## Methods

### Clinical populations and sample collection

With the written consent of the parents and the approval (No. 2016016) of the Ethics Committee of our institute, all umbilical cord specimens were obtained immediately from the newborn by cesarean operation at the Zhongnan Hospital of Wuhan University and collected in sterile boxes containing normal saline.

### Enzyme-linked immunosorbent assay (ELISA)

The concentrations of serum cortisol were measured by ELISA kit (R&D, Minneapolis, MN, USA), following the manufacturer’s protocols.

### Isolation and culture of human WJ-MSCs

Human WJ-MSCs were isolated as previously described [40]. Briefly, MSCs were isolated from collected human umbilical cords within 2 h. Removing the umbilical arteries and umbilical vein, Wharton’s jelly was peeled off from the remaining part of the umbilical cords and transferred to a sterile container and then cut into pieces smaller than 0.5 cm^3^. The minced Wharton’s jelly was digested for 4 h in a 50-ml sterile centrifuge tube with 30-ml culture medium containing collagenase of type I (Invitrogen, Thermo Fisher Scientific Inc., USA) at 0.2% in an incubator (5% carbon dioxide, 37 °C). After centrifuging the liquid at 300×*g* for 15 min and discarding the supernatants, the cells were resuspended in DMEM/F12 medium (Gibco BRL, Thermo Fisher Scientific Inc., USA) with 10% fetal bovine serum (Gibco BRL, Thermo Fisher Scientific Inc., USA) and 1% penicillin-streptomycin (Gibco BRL, Thermo Fisher Scientific Inc., USA) in humidified air with 5% carbon dioxide at 37 °C. The WJ-MSCs were passaged once the flask reached approximately 80% confluence and the fourth passage was used for the next experiments.

### Characterization of WJ-MSCs by flow cytometry

Flow cytometry was utilized to determine the stemness features of WJ-MSCs by analysis of specific cell surface markers. After being trypsinized, the cells were resuspended in 0.5 ml phosphate-buffered saline (PBS) and incubated for 1 h at room temperature with conjugated primary antibodies (FITC-CD34, CD45, CD73, CD90 and CD105, eBioscience, San Diego, CA, USA) and resuspended in 0.5 ml PBS and then analyzed using a BD FACS Canto flow cytometer (Becton Dickinson).

### Establishment of two-step cell model and cell treatment

WJ-MSCs were cultured in alginate beads following the modified method described by De Ceuninck et al. [41]. Briefly, WJ-MSCs cultured in monolayer were trypsinized, washed, and centrifuged. Then, the WJ-MSCs were suspended at a concentration of 3 × 10^6^ cells/ml in a 1.25% alginate (Sigma-Aldrich, St. Louis, MO, USA) in 0.15 M NaCl and slowly dropped into 102 mM CaCl_2_ solution to form alginate beads. The beads were cultured with a chondrogenic medium: DMEM/F12 medium containing 1% insulin-transferrin-selenous (ITS) (Sigma, St. Louis, MO, USA), 100 nM dexamethasone (Sigma, St. Louis, MO, USA), and 10 ng/ml transforming growth factor β1 (TGFβ1) (Sigma, St. Louis, MO, USA). After chondrogenic differentiation of 3 weeks, some beads were collected for detection. The remaining beads in the plates were treated with DMEM/F12 medium containing 10 ng/ml recombinant human interleukin-1β (rhIL-1β, Prop Tech, London, UK) for 24-h and then collected for analysis.

In the experiment of differentiation and IL-1β induction, all the specimens were divided into three groups, namely the control, IUGR, and cortisol-treated groups, among which, the control group refers to WJ-MSCs from normal newborns without cortisol treatment, the IUGR group refers to WJ-MSCs from IUGR newborns without cortisol treatment, and the cortisol-treated groups refer to WJ-MSCs from normal newborns treated by different concentration of cortisol, including 300 and 1200 nM. RU486 (10 μM) (Sigma-Aldrich, St. Louis, MO, USA) and LMK235 (100 nM) (Sigma-aldrich, St. Louis, MO, USA) were respectively utilized with different concentrations of cortisol (300 and 1200 nM) to treat the WJ-MSCs during chondrogenic differentiation in a 6-well culture plate.

### Cell viability analysis

After 21-day differentiation of WJ-MSCs, 8 alginate beads were randomized taken to 96-well plate and were given 50 μL basic culture media and 20 μL MTS solution (Promega, USA) to incubate for 2 h; after that, the alginate beads were dissolved by beads solution (containing 12 mg/mL NaCl, 16.20 mg/mL trisodium citrate dehydrate, 2.4 mg/mL HEPES) for 1 min, and finally mix the cell suspension well. Then, 490-nm wavelength was selected to determine the absorption value of various apertures at the GENios VA200 enzyme standard (TECAN, Austria), and the results were recorded.

### Alcian blue and safranin-O staining of alginate beads

After differentiation of human WJ-MSCs and IL-1β induction, 3 beads in each group were harvested and fixed in 10% buffered paraformaldehyde at room temperature. Then, these beads were rinsed with phosphate-buffered saline (PBS), serially dehydrated, infiltrated with arnyl acetate, paraffin embedded, and sectioned at 5-μm thickness for staining [42]. In detail, the sections were rinsed with PBS and then stained overnight with 1% Alcian blue dye at pH 1.0 or 0.1% aqueous safranin-O for 10 min at room temperature. Images were captured with an Olympus AH-2 light microscope (Olympus, Tokyo, Japan) and quantitatively analyzed with ImageJ software (National Institutes of Health, Bethesda, MD) using methodology as previously described [43, 44]. Images were made binary under an RGB threshold, and “Particle Analysis” was utilized to measure the positive area and normalized to the control group.

### Total RNA extract and RT-qPCR

Total RNA was isolated from the collected alginate beads and rat knee cartilage, using Trizol reagent (Invitrogen, Thermo Fisher Scientific Inc., USA) following the manufacturer’s protocol. The concentration and purity of the isolated RNA were determined by spectrophotometer and adjusted to 1 μg/μL. Total RNA was stored in diethyl pyrocarbonate-H_2_O (DEPC-H_2_O) at − 80 °C. For RT-qPCR analysis, single-strand cDNA was prepared from 2 μg of total RNA according to the protocol of the Exscript RT reagent kit. Primers were designed using Primer Premier 5.0 and their sequences are shown in Table 1. PCR assays were performed in 384-well optical reaction plates using the RG-3000 Rotor-Gene 4 Channel Multiplexing System (Corbett Research Pty Ltd., Sydney, Australia) in a total volume of 25 μL reaction mixture containing 2 μL of 0.1 μg/μL cDNA template, 0.5 μL of 10 μmol/L each primer, 12.5 μL of 2 × Premix Ex Taq, 0.5 μL of 20 × SYBR Green I, and 9 μL of DEPC-H_2_O. To precisely quantify the transcript expression of these genes including α1 chain of type II collagen (*COL2A1*), aggrecan (*ACAN*), transforming growth factor β receptor I (*TGFβRI*), matrix metalloproteinase 3 (*MMP3*), *MMP13*, a disintegrin and metalloprotease with thromospondinmotifs 5 (*ADAMTS5*) and histone deacetylation (*HDAC*), the mRNA level of glyceraldehyde phosphate dehydrogenase (*GAPDH*) was measured as the quantitative control, and each sample was normalized on the basis of GAPDH mRNA content. PCR cycling conditions were as follows: 95 °C, 15 s for pre-denaturation, and 95 °C, 5 s for denaturation; annealing conditions for each gene are listed in Table 1.

**Table 1:** Oligonucleotide primers used for RT-qPCR conditions

Genes	Forward primer	Reverse primer	Annealing
Homo *GAPDH*	GAAATCCCATCACCATCTTCCAG	GAGTCCTTCCACGATACCAAAG	60
Homo *COL2A1*	GCTCCCAGAACATCACCTACCA	ACAGTCTTGCCCCACTTACCG	60
Homo *ACAN*	AAGGGCGAGTGGAATGATGT	CGCTTCTGTAGTCTGCGTTTGT	60
Homo *TGFβRI*	GCAATGGGCTTAGTATTCTGGG	TCCTGTTGACTGAGTTGCGATAAT	60
Homo *Smad2*	TCTGGGCAGCCGTAAGTTTA	CCACTGTTGCGACGATTAGG	60
Homo *Smad3*	CGGTTCACAAGGCTCAAGAG	AAGTGGGTCCTCAGAAGTGG	60
Homo *MMP3*	AATCAATTCTGGGCTATCAGAGG	GCATCAATCTTTGAGTCAATCCC	60
Homo *MMP13*	CAGAACTTCCCAACCGTATTGAT	TGTATTCAAACTGTATGGGTCCG	60
Homo *ADAMTS5*	TTTCTCCAAAGGTGACCGATG	CCTCCACATACTCCGCACTTG	60
Rat *GAPDH*	GCAAGTTCAACGGCACAG	GCCAGTAGACTCCACGACA	60
Rat *TGFβRI*	CTCGAGCAGTTACAAAGGGC	CTCGAGCAGTTACAAAGGGC	60

*GAPDH*, glyceraldehyde phosphate dehydrogenase; *COL2A1*, α1 chain of type II collagen; *ACAN*, Aggrecan; *TGFβRI*, transforming growth factor β receptor I; *MMP3*, matrix metalloproteinase 3; *MMP13*, matrix metalloproteinase 13; *ADAMTS5*, a disintegrin and metalloprotease with thromospondinmotifs 5

### Chromatin immunoprecipitation (ChIP) assay

Cells in Alginate beads were cross-linked with 1% formaldehyde before sonicating in SDS lysis buffer. DNA in cell lysates was sheared to length of approximately 200 base pairs. Fragmented chromatin was first pre-cleared with protein A-sepharose 4B and rabbit IgG for 2 h. Before immunoprecipitating with fresh protein A-sepharose 4B and antibody include anti-histone 3 lysine 9 acetylation (H3K9ac) and anti-H3K27ac (Abcam, USA) at 4 °C overnight. Sepharose beads were washed before eluting with 1% SDS followed by reverse cross-linking at 65 °C overnight. The samples were then placed in a 65 °C water bath overnight to reverse formaldehyde cross-linking and subsequently were purified using PCR purification kits. The isolated DNA was then assayed using RT-qPCR; the primer sequences of the promoters of indicated genes are shown in Table 2. The input values were compared to the immunoprecipitated samples, with the IgG negative controls values subtracted as background. The calculated errors in all of the graphs depicting ChIP data represent the standard deviations for three replicate RT-qPCRs for precipitated chromatin, input chromatin, and background (i.e., chromatin precipitated with nonspecific IgG).

**Table 2:** Oligonucleotide primers and PCR conditions for CHIP-PCR

Genes	Forward primer	Reverse primer	Annealing
Homo *COL2A1*	TGCAGGGAAGGGCTAAAAGA	GGAGCCCACAGAGATTCAGA	60
Homo *ACAN*	CTCGAACTCAGTCCCACCTT	ACCTGCCCCTAACCAAAGAT	60
Homo *TGFβRI*	ATCGGGAAGGGGTTTGAGAG	AGATCCTGAGCCCAAACACA	60
Homo *Smad2*	CGAGTGCCTAAGTGATAGT	AGACTGAGCCAGAAGAGC	60
Homo *Smad3*	GGGCTTTGAGGCTGTCTA	CCAACCCGATCCCTTTAC	60
Rat *TGFβRI*	ACTGGAATTTGAGGAGGGCA	TAGACCCGCTCCTCAATTCC	60

### Western blotting

To obtain protein, the cells were harvested and dissolved in RIPA buffer. Protein concentrations were determined by BCA protein assay kit. Equal amounts of protein lysates (30 μg/lane) were loaded and resolved on 10% SDS polyacrylamide gel and then transferred onto nitrocellulose filter, and probed with rabbit anti-TGFβRI (1:1000, Abcam, Inc., UK), COL2A1 (1:500, Abcam, Inc., UK), ACAN (1:1000, Abcam, Inc., UK), and GAPDH (1:1000, Abcam, Inc., UK) at 4 °C overnight. After incubation with horse radish peroxidase-conjugated secondary antibody, blots were developed by enhanced chemiluminescence following the manufacturer’s protocol and visualized by exposure to a Fusion FX system (Vilber Lourmat, Marne-la-Vallée, France). Protein amount in electrophoresis gel was analyzed with Quantity One 4.6 analysis software (Bio-Rad Laboratories Inc., CA, USA).

### Establishment of animal models

The animal experiment was performed in the Center for Animal Experiment of Wuhan University (Wuhan, China), which has been accredited by the Association for Assessment and Accreditation of Laboratory Animal Care International (AAALAC International). The protocol was approved by the Committee on the Ethics of Animal Experiments of the Wuhan University School of Medicine (Permit Number: 14016). All animal experimental procedures were performed in accordance with the Guidelines for the Care and Use of Laboratory Animals (eighth edition) by the National Research Council of the United States National Academies.

#### In utero

Specific pathogen-free (SPF) Wistar rats, females weighing 200–240 g and males weighing 260–300 g, were obtained from the Experimental Center of Hubei Medical Scientific Academy (No. 2009-0004, Hubei, China). Animals were housed (room temperature 18–22 °C; humidity 40–60%), acclimated, and mated. Upon confirmation of mating by the appearance of sperm in a vaginal smear, the day was taken as gestational day (GD) 0. Pregnant females were then transferred to individual cages. Pregnant rats were randomly divided into two groups: the control group, and prenatal xenobiotics exposure (PXE) group. Starting from GD9 until GD20, the PXE group were administrated of caffeine (120 mg/kg/d), nicotine (2 mg/kg/d), ethanol (4 g/kg/d), or dexamethasone (0.2 mg/kg/d) as previously described [19–22], while the control group was given the same volume of distilled water. On GD20, 8 randomly selected pregnant rats with 10–14 live fetuses from each group were anesthetized. The male fetuses were quickly removed, weighed, and IUGR was diagnosed when the body weight of a fetus was two standard deviations less than the mean body weight of fetuses in the control group. Fetal knee joints were separated under a dissecting microscope and collected from each littermate were pooled together and immediately frozen in liquid nitrogen, followed by storage at − 80 °C for analyses. A portion of fetal knee joints (one per litter) were fixed in 4% paraformaldehyde for morphological observation.

#### After birth

On the postnatal week (PW) 1, we weighed the litter sizes and recorded the weight gain. The pups in each litter were randomly divided into 4 batches, according to the postnatal week respectively named as PW6. For each batch, 10 male pups for control or PXE group were selected randomly, and all of the pups were weaned to an ad libitum diet before being sacrificed. On PW6, the corresponding batches of rats were anesthetized with ether and decapitated to collect knee tissues. For histological analysis, the knee joints were fixed in 4% paraformaldehyde for 3 days, decalcified in 20% EDTA (pH 7.4) for 21 days, and embedded in paraffin. Serial 5-μm-thick sagittal sections were cut across the whole joint. The remaining knee samples were used for RT-qPCR and ChIP-PCR.

### Immunofluorescence staining of cartilage samples

Sections were deparaffinized in xylene and hydrated through a graded series of alcohols. Hydrated sections were then applied for immunofluorescence. Briefly, after antigen retrieval with boiling in sodium citrate buffer, sections were then blocked in serum for 30 min followed by incubation with the primary antibody rabbit anti-TGFβRI (Abcam, Inc., UK) in a humidified chamber at 4 °C overnight. Following washing with PBS, the sections were incubated with Alexa Fluor 594-labeled secondary antibody (red color) (1:100) (Proteintech) in darkness at room temperature for 2 h. Nuclei were counterstained with DAPI (blue color) in darkness for 5 min. The staining was examined using an Olympus AH-2 light microscope (Olympus, Tokyo, Japan).

### Statistical analysis

SPSS 17 (SPSS Science Inc., Chicago, IL) was used for data analysis. Quantitative data were expressed as the mean ± S.E.M. and were evaluated with an independent samples *t* test or using one-way ANOVA followed by Dunnett’s post hoc Student’s *t* tests. Statistical significance was defined as *P* < 0.05.

## Results

### Poor chondrogenic differentiation of WJ-MSCs from IUGR humans and subsequent susceptibility to the osteoarthritis-like phenotype upon IL-1β stimulation

**Fig. 1:**
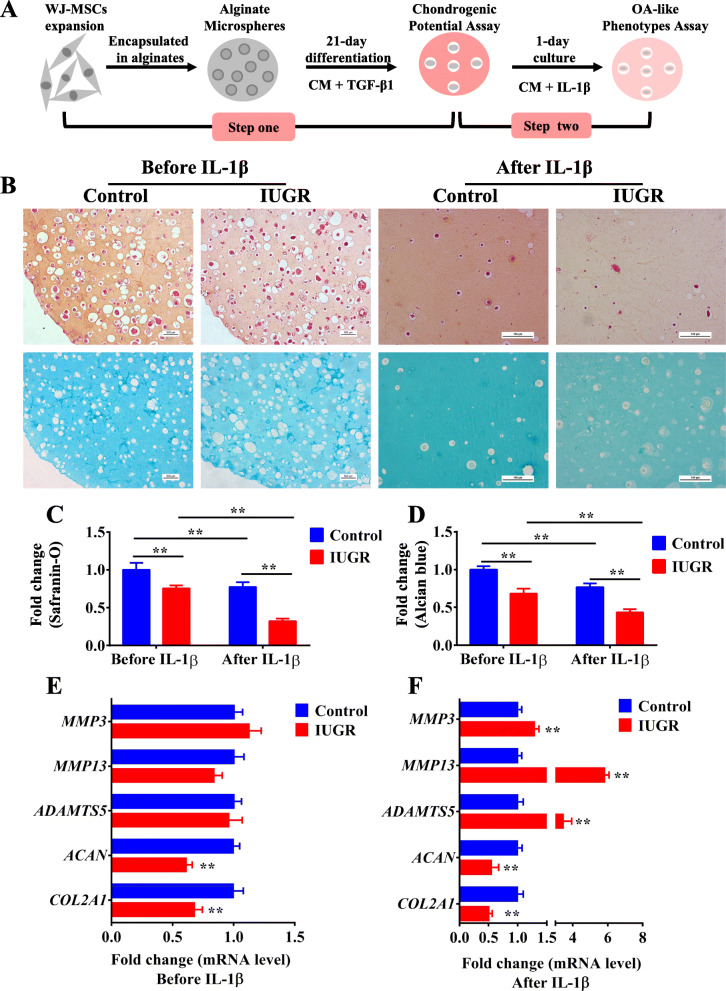
Poor chondrogenic differentiation of WJ-MSCs from IUGR humans and subsequent increased susceptibility to an osteoarthritis-like phenotype induced by IL-1β. **a** A schematic of a two-step cell culture model for evaluating chondrogenic differentiation and susceptibility to an osteoarthritis-like phenotype. **b** Safranin-O and Alcian blue staining for glycosaminoglycan in WJ-MSCs after chondrogenic differentiation for 21 days and IL-1β treatment for 1 day in the control and IUGR groups. **c**, **d** Relative quantification of Safranin-O and Alcian blue staining, *n* = 5. **e**, **f** RT-qPCR analysis of *COL2A1*, *ACAN*, *MMP3*, *MMP13*, and *ADAMTS5* expression in WJ-MSCs after chondrogenic differentiation and IL-1β treatment in the control and IUGR groups, *n* = 5. WJ-MSCs, Wharton’s jelly-derived mesenchymal stem cells; IUGR, intrauterine growth retardation; IL-1β, interleukin-1β; CM, chondrogenic medium; TGFβ1, transforming growth factor β1; RT-qPCR, real-time quantitative polymerase chain reaction; *COL2A1*, α1 chain of type II collagen; ACAN, aggrecan; *MMP*, matrix metalloproteinase; *ADAMTS5*, a disintegrin and metalloproteinase with thrombospondin motifs-5. Data are the mean ± S.E.M. **P < 0.01 *vs* control

To mimic the processes of cartilage development in utero and the inflammation during osteoarthritis process in vivo, we constructed a two-step cell culture model based on the human WJ-MSCs from IUGR individuals. Firstly, WJ-MSCs were obtained from the umbilical cord of the newborns diagnosed as IUGR [45]. Then, step one: WJ-MSCs from normal and IUGR individuals were induced to differentiate into chondrocytes in an alginate scaffold for 21 days, a standard process of chondrogenic differentiation in vitro [33]. Step two: the chondrogenic WJ-MSCs were treated with 10 ng/ml IL-1β [46] for 24 h to evaluate their susceptibility to an osteoarthritis-like phenotype (Fig. 1a). Flow cytometry was performed to identify the 3rd generation of human WJ-MSCs (Fig. S1) as previously reported [47]. After chondrogenic differentiation, compared with those in the control group, the cell viability of the IUGR group had no significant change (Fig. S2A), Safranin-O and Alcian blue staining showed that glycosaminoglycan contents in the alginate were decreased (*P* < 0.01, Fig. 1b–d), and the mRNA expression levels of the phenotypic genes *COL2A1* and *ACAN* were significantly decreased (*P <* 0.01, Fig. 1e), but the mRNA expression levels of catabolic factors, including *MMP3*, *MMP13*, and *ADAMTS5* were not changed (Fig. 1e). After IL-1β treatment, the glycosaminoglycan content and the expression of the phenotypic genes in the IUGR group were decreased more severely (*P <* 0.01, Fig. 1b–d, f), while the mRNA expression levels of *MMP3*, *MMP13*, and *ADAMTS5* were dramatically increased (*P <* 0.01, Fig. 1e). All the above results suggested that WJ-MSCs from IUGR newborns had a poor capacity for chondrogenic differentiation and the subsequent differentiated chondrocytes were more susceptible to an osteoarthritis-like phenotype.

### Poor chondrogenic differentiation of normal WJ-MSCs induced by excessive cortisol and subsequent susceptibility to an osteoarthritis-like phenotype upon IL-1β stimulation

**Fig. 2:**
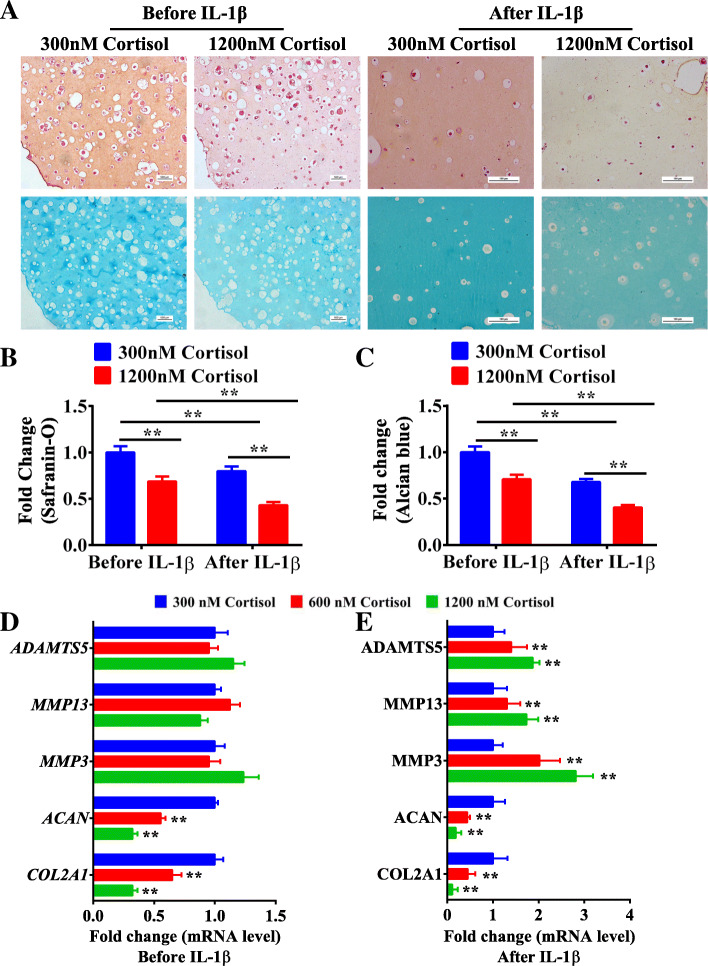
Normal WJ-MSCs treated with high levels of cortisol presented a poor capacity for chondrogenic differentiation and subsequent increased susceptibility to an osteoarthritis-like phenotype induced by IL-1β. **a** Safranin-O and Alcian blue staining for glycosaminoglycan in WJ-MSCs after chondrogenic differentiation for 21 days and IL-1β treatment for 1 day in 300, 600, and 1200 nM cortisol groups. **b**, **c** Relative quantification of Safranin-O and Alcian blue staining, *n* = 5. **d**, **e** RT-qPCR analysis of *COL2A1*, *ACAN*, *MMP3*, *MMP13*, and *ADAMTS5* expression after chondrogenic differentiation and IL-1β treatment in 300, 600, and 1200 nM cortisol groups, *n* = 5. WJ-MSCs, Wharton’s jelly-derived mesenchymal stem cells; RT-qPCR, real-time quantitative polymerase chain reaction; *COL2A1*, α1 chain of type II collagen; *ACAN*, aggrecan; *MMP*, matrix metalloproteinase; *ADAMTS5*, a disinterring and metalloproteinase with thrombospondin motifs-5. Data are the mean ± S.E.M. **P < 0.01 vs control

To investigate whether maternal cortisol overexposure is the initial factor involved in these outcomes, we first detected concentrations of cortisol in the neonatal umbilical cord blood. The result showed that the cortisol level in samples from the IUGR group was significantly higher than the newborns with normal birthweight (*P <* 0.01, Fig. S3), which was consistent with the result reported by Mericq et al. [48]. Taking the reported data and our present results into account, we chose 300 nM cortisol as the physiological concentration and 600 nM and 1200 nM as a series of pathological concentrations in vitro. Then, the chondrogenic potential of WJ-MSCs treated with different concentrations of cortisol and the subsequent susceptibility to an osteoarthritis-like phenotype were evaluated. Compared with the 300 nM cortisol group, the cell viability in the 600 and 1200 nM cortisol groups had no significant changes on 0 day and 21th day after chondrogenic differentiation (Fig. S2B), while the glycosaminoglycan staining in the 1200 nM cortisol group was significantly decreased (*P* < 0.01, Fig. 2a–c). The mRNA expression levels of *COL2A1* and *ACAN* in the 600 and 1200 nM groups were substantially reduced (*P <* 0.01, Fig. 2d), while the mRNA expression levels of *MMP3*, *MMP13*, and *ADAMTS5* were not changed (Fig. 2d). After IL-1β treatment, the glycosaminoglycan staining (*P* < 0.01, Fig. 2a–c) and mRNA levels of *COL2A1* and *ACAN* in the 1200 nM cortisol group were decreased more markedly (*P* < 0.01, Fig. 2e). Simultaneously, the mRNA levels of *MMP3*, *MMP13*, and *ADAMTS5* were significantly enhanced (*P <* 0.01, Fig. 2e). All the above results suggested that normal WJ-MSCs treated with excessive cortisol presented an insufficient chondrogenic differentiation capacity and the subsequent differentiated chondrocytes were more susceptible to an osteoarthritis-like phenotype.

### Decreased H3K9ac level of *TGFβRI* participated in the poor chondrogenic differentiation of human WJ-MSCs induced by excessive cortisol

**Fig. 3:**
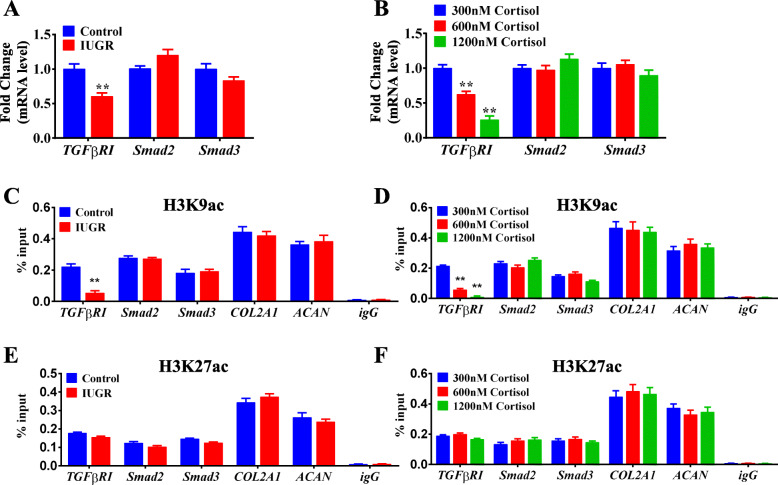
Decreased H3K9ac level of *TGFβRI* mediated the poor chondrogenic differentiation of WJ-MSCs. **a** RT-qPCR analysis of TGFβRI, Smad2, and Smad3 after chondrogenic differentiation in the control and IUGR groups. **b** RT-qPCR analysis of *TGFβRI*, *Smad2*, and *Smad3* after chondrogenic differentiation in the 300, 600, and 1200 nM cortisol groups. *n* = 5. **c**, **e** ChIP-PCR analysis of H3K9ac and H3K27ac levels of *TGFβRI*, *Smad2*, and *Smad3*, *COL2A1* and *ACAN* after chondrogenic differentiation in the control and IUGR groups. *n* = 3. **d**, **f** ChIP-PCR analysis of H3K9ac and H3K27ac of *TGFβRI*, *Smad2*, and *Smad3*, *COL2A1* and *ACAN* after chondrogenic differentiation in the 300, 600, and 1200 nM cortisol groups. *n* = 3. H3K9ac, histone 3 lysine 9 acetylation; H3K27ac, histone 3 lysine 27 acetylation; RT-qPCR, real-time quantitative polymerase chain reaction; *TGFβRI*, transforming growth factor β receptor I; *COL2A1*, α1 chain of type II collagen; *ACAN*, aggrecan; WJ-MSCs, Wharton’s jelly-derived mesenchymal stem cells; IUGR, intrauterine growth retardation; ChIP-PCR, chromatin immunoprecipitation-polymerase chain reaction. Data are Mean ± S.E.M. **P < 0.01 vs control

To explore the potential pathway involved in the poor chondrogenic differentiation of WJ-MSCs from IUGR, we focused on the TGFβ signaling pathway, which has been reported to be indispensable for the chondrogenic differentiation of mesenchymal stem cells (MSCs) both in vivo and in vitro [40, 49, 50]. The results showed that the mRNA expression of *TGFβRI* was lower in the chondrogenic WJ-MSCs from IUGR individuals than that in the individuals with normal birthweight (*P <* 0.01, Fig. 3a), while the mRNA expression levels of *Smad2* and *Smad3* were unaffected. Moreover, similar results were observed in the groups treated with excessive cortisol (*P <* 0.01, Fig. 3b). Such findings indicated that the poor chondrogenic differentiation of WJ-MSCs might be attributed to the decreased expression of *TGFβRI*.

Growing evidence indicates that excessive glucocorticoid has powerful effects on the epigenome to influence gene expression [28, 29]. Prenatal glucocorticoid exposure in a fetal guinea pig model resulted in an acute and substantial effect on the histone acetylation of target genes in the hippocampus [51]. Accordingly, we hypothesized that excessive cortisol might inhibit the expression of *TGFβRI* through histone acetylation modification. To verify this hypothesis, ChIP-PCR was performed to examine the histone acetylation level of the *TGFβRI* in the differentiated cells. Interestingly, compared to the control group, the H3K9ac level of *TGFβRI* in the IUGR group (*P <* 0.01, Fig. 3c) and excessive cortisol-treated groups (*P <* 0.01, Fig. 3d) was decreased dramatically. However, the H3K27ac levels of *TGFβRI* were not changed (Fig. 3e, f). Additionally, the H3K9ac and H3K27ac levels of *Smad2*, *Smad3*, *COL2A1*, and *ACAN* did not differ among the groups (Fig. 3c–f). These results suggested that the reduced H3K9ac level of *TGFβRI* in human WJ-MSCs was induced by excessive cortisol, which resulted in the decreased expression of *TGFβRI* and further contributed to the poor chondrogenic differentiation in vitro.

### GR/HDAC4 participated in the reduced H3K9ac level of *TGFβRI* induced by excessive cortisol

**Fig. 4:**
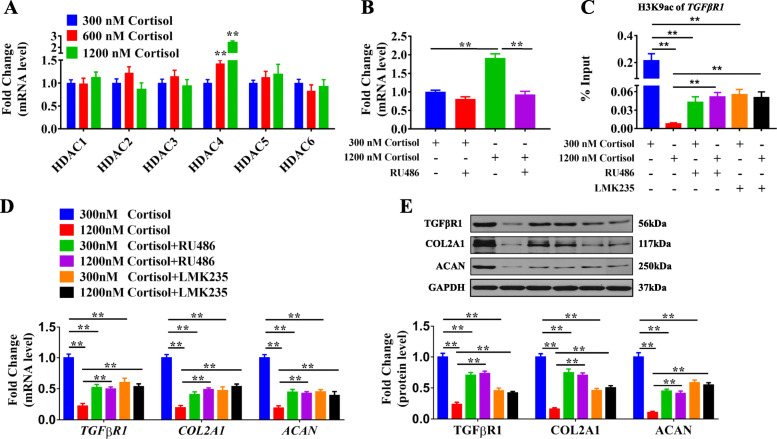
GR/HDAC4 mediated the H3K9 deacetylation of *TGFβRI* induced by high levels of cortisol. **a** RT-qPCR analysis of *HDACs* expression in the WJ-MSCs treated with 300, 600, and 1200 nM cortisol after chondrogenic differentiation for 7 days. *n* = 5. **b** RT-qPCR analysis of *HDAC4* expression in WJ-MSCs treated with cortisol and RU486 (10 μM) after chondrogenic differentiation for 7 days. *n* = 5. **c** ChIP-PCR analysis of the H3K9ac level of *TGFβRI* in WJ-MSCs treated with cortisol, and RU486 (10 μM) or LMK235 (100 nM) after chondrogenic differentiation for 7 days. *n* = 3. **d** RT-qPCR analysis of *TGFβRI*, *COL2A1*, *and ACAN* expression in WJ-MSCs treated with cortisol and RU486 (10 μM) or LMK235 (100 nM) after chondrogenic differentiation for 7 days. *n* = 5. **e** Western blot analysis of TGFβRI, COL2A1, and ACAN in WJ-MSCs treated with cortisol, RU486 (10 μM), or LMK235 (100 nM) after chondrogenic differentiation for 7 days, *n* = 5. RT-qPCR, real-time quantitative polymerase chain reaction; *GR*, glucocorticoid receptor; *HDAC4*, histone deacetylase 4; H3K9ac, histone 3 lysine 9 acetylation; *TGFβRI*, transforming growth factor β receptor I; WJ-MSCs, Wharton’s jelly-derived mesenchymal stem cells; ChIP-PCR, chromatin immunoprecipitation-polymerase chain reaction; igG, immunoglobulin G. Data are the mean ± S.E.M. **P < 0.01 vs control

It has been reported that glucocorticoid regulates the expression of many target genes through the glucocorticoid receptor (GR) and epigenetic enzymes, such as HDACs [52, 53]. Thus, to elucidate the potential mechanism of H3K9ac of *TGFβRI* induced by excessive cortisol, we detected the expression of *HDACs* in WJ-MSCs. The results showed that the mRNA expression of *HDAC4* was significantly increased in a concentration-dependent manner in the cortisol groups of 600 nM and 1200 nM compared with that in the 300 nM cortisol group (*P* < 0.01, Fig. 4a). Then, RU486 (a GR antagonist) and LMK235 (a specific HDAC4 inhibitor) were utilized to verify the roles of GR and HDAC4. The results showed that, compared with the 300 nM cortisol group, RU486 attenuated the increase of expression of *HDAC4* induced by 1200 nM cortisol (*P* < 0.01, Fig. 4b). Furthermore, RU486 and LMK235 not only abolished the decrease in the H3K9ac level of *TGFβRI* induced by cortisol of 1200 nM (*P* < 0.05, *P* < 0.01, Fig. 4c), but also reversed the reduced mRNA and protein levels of *TGFβRI*, *COL2A1*, and *ACAN* (*P* < 0.01, Fig. 4d, e). The results above suggested that GR, combined with HDAC4, participated in the decreased H3K9ac level of *TGFβRI* induced by excessive cortisol, which led to the closed chromatin in the *TGFβRI* promoter region and further resulted in the decline in the transcriptional expression of *TGFβRI*.

### Decreased H3K9ac and expression levels of *TGFβRI* were verified in IUGR individuals

**Fig. 5:**
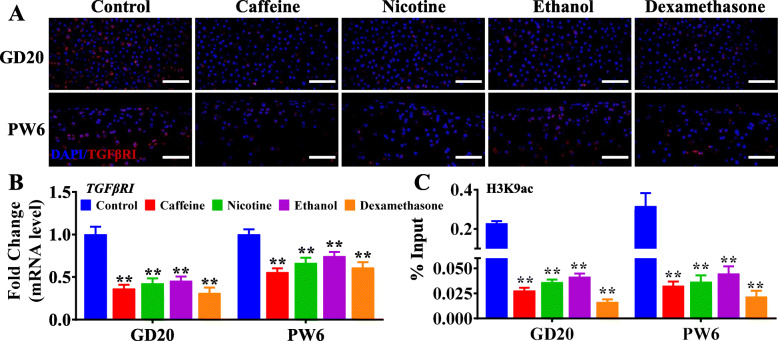
Decreased expression and H3K9ac levels of *TGFβRI* were verified in the rats with low birth weight. **a** Immunofluorescence analysis of TGFβRI in the cartilage of male IUGR rat offspring caused by prenatal caffeine, nicotine, ethanol, and dexamethasone exposure, scale bar = 50 μm. **b** RT-qPCR analysis of *TGFβRI* in the cartilage of male offspring with IUGR caused by prenatal caffeine, nicotine, ethanol, and dexamethasone exposure. *n* = 6. **c** ChIP-PCR analysis of *TGFβRI* in the cartilage of male offspring with IUGR caused by prenatal caffeine, nicotine, ethanol, and dexamethasone exposure. *n* = 3. GD, gestational day; PW6, postnatal week 6; DAPI, 4′,6-diamidino-2-phenylindole; RT-qPCR, real-time quantitative polymerase chain reaction; TGFβRI, transforming growth factor β receptor I; ChIP-PCR, chromatin immunoprecipitation-polymerase chain reaction. Data are the mean ± S.E.M. ^#^*P <* 0.05, ^##^*P <* 0.01 vs before the second hit, namely intra-articular papain injection, strenuous running, or ovariectomy; *P < 0.05, **P < 0.01 vs control

**Fig. 6:**
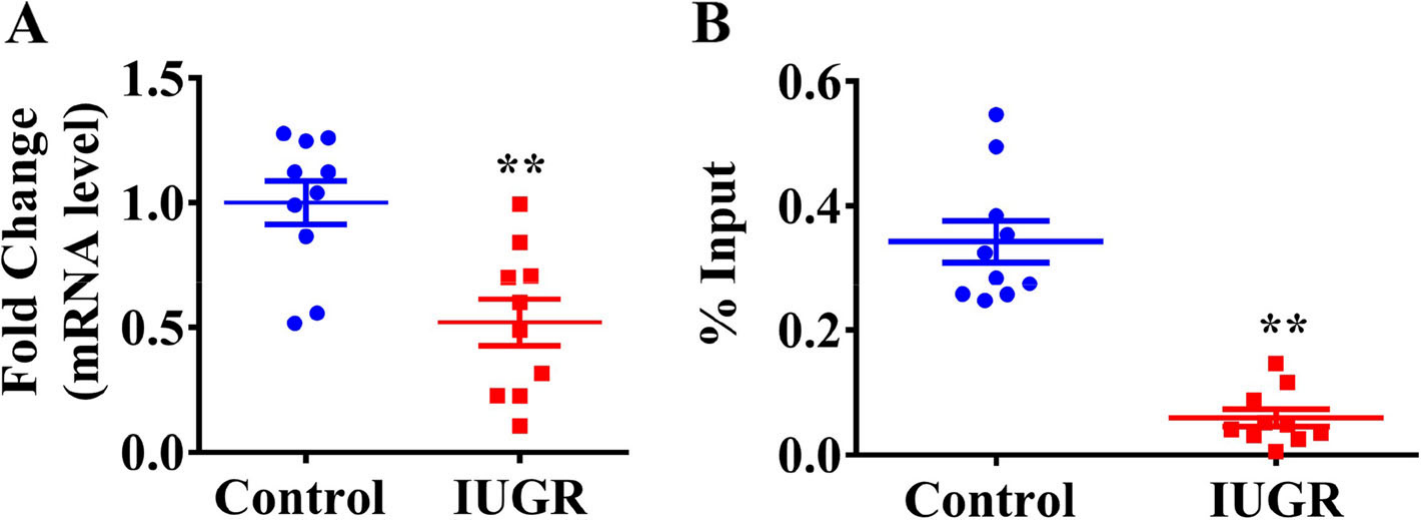
Decreased expression and H3K9ac levels of *TGFβRI* were verified in the umbilical cord from IUGR individuals. **a** RT-qPCR analysis of *TGFβRI* expression in the umbilical cord from IUGR individuals, *n* = 10 in the control group, *n* = 10 in the IUGR group. **b** ChIP-PCR analysis of *TGFβRI* promoter in the umbilical cord from IUGR, *n* = 10 in the control group, *n* = 10 in the IUGR group. TGFβRI, transforming growth factor β receptor I; IUGR, intrauterine growth retardation; RT-qPCR, real-time quantitative polymerase chain reaction; ChIP-PCR, chromatin immunoprecipitation-polymerase chain reaction. Data are the mean ± S.E.M. *P < 0.05, **P < 0.01 vs control

Our laboratory previously confirmed that IUGR rats induced by caffeine, nicotine, ethanol, and dexamethasone during pregnancy developed persistent chondrodysplasia and susceptibility to osteoarthritis in adulthood [19–22]; thus, we detected the H3K9ac level of TGFβRI and its expression in the cartilage of IUGR rat offspring. The results showed that the H3K9ac level of TGFβRI and its mRNA and protein expression were decreased dramatically both in utero and at postnatal week 6 in the offspring rats with prenatal caffeine, nicotine, ethanol, and dexamethasone exposure (*P* < 0.01, Fig. 5a–c). Furthermore, we collected the WJ-MSCs from the umbilical cord of human newborns and found that H3K9ac level of TGFβRI and its mRNA were all lower in the human newborns with low birthweight, when compared with those with normal birthweight (*P* < 0.01, Fig. 6a, b).

## Discussion

### Poor chondrogenic differentiation of WJ-MSCs from IUGR humans and the subsequent increased susceptibility to the osteoarthritis-like phenotype were confirmed via a two-step cell culture model

During the process of chondrogenesis of MSCs, TGFβ1 in the medium induces the activation of a transmembrane heteromeric complex of serine/threonine kinases including TGFβRI [54]. Following the phosphorylation of the TGFβR, Smad2, and Smad3 associate with Smad4 and then this complex migrates into the nucleus and participates in the transcriptional activation of the chondrogenic genes [55]. In the present study, after chondrogenic differentiation, the glycosaminoglycan content, as measured by Safranin-O and Alcian blue staining, and the expression levels of phenotypic genes, including *COL2A1* and *ACAN*, as well as the expression of *TGFβRI*, were decreased in the IUGR group, suggesting that the low expression of *TGFβRI* led to the reduced responsiveness to TGFβ1 and further resulted in the poor differentiation of MSCs into chondrocytes.

It has been reported that articular cartilage is mainly formed in embryonic and neonatal periods. After maturation, hardly any proliferation occurs in the chondrocyte. Furthermore, cartilage dysplasia is one of the risk factors of osteoarthritis [56, 57]. Therefore, the developmental level of articular cartilage in utero influences the quality of cartilage in adulthood [58, 59]. Based on our previous studies [14–22, 60, 61], we propose a “two-hit” theory about the fetal origination of adult osteoarthritis. More specifically, the adverse prenatal environment as the first hit induces persistent cartilage dysplasia and attenuates the resistance of cartilage to local inflammation in adulthood in the IUGR offspring. Then, an inflammatory microenvironment induced by aging, obesity-related lipid factors, and inappropriate mechanical factors [62], as the second hit, could augment the susceptibility to adult osteoarthritis. It has been revealed that IL-1β is the major inflammatory mediator during the osteoarthritis process [63]. In articular cartilage, the effects of TGFβ1 are counterbalanced by the action of IL-1β [64, 65], which further indicates that the decreased activity of the TGFβ pathway might result in the reduced resistance of the cartilage in response to inflammation. In the differentiated chondrocytes from the IUGR group treated with IL-1β, the glycosaminoglycan content and the expression of phenotypic genes were decreased more severely. Moreover, the expression of catabolic factors, including *MMP3*, *MMP13*, and *ADAMTS5*, was greatly enhanced. These results demonstrated that the poorly differentiated chondrocytes were more susceptible to an osteoarthritis-like phenotype after IL-1β stimulation.

### Maternal glucocorticoids overexposure and the subsequent decreased H3K9ac level of *TGFβRI* played a critical role in the poor chondrogenic differentiation of WJ-MSCs from IUGR individuals

Baseline glucocorticoids level plays a crucial role in determining the maturation of fetal tissues [25]. A clinical study had reported a significant increase in cortisol level in the umbilical blood of IUGR individuals [66]. The cortisol level (range 221~801 nM) in the umbilical blood of IUGR individuals was found significantly higher than that of the normal infants (121~395 nM) [48]. In the present study, our results showed that the serum cortisol concentration ranged from 121~1538 nM in the IUGR individuals and from 21~369 nM in the normal individuals, which was consistent with the previous findings. Based on the above data, 300 nM cortisol was set as the physiological concentration, while 600 and 1200 nM were set as the pathological concentrations as the excessive maternal glucocorticoids.

An increasing number of studies have suggested that glucocorticoids are involved in intrauterine programming through epigenetic modifications, which could be inherited by the next generation [67]. Our present results further confirmed the programming effects of glucocorticoids and their potential molecular mechanism. This view was supported by our present evidence including (i) the serum cortisol level in the human IUGR umbilical blood was increased; (ii) normal human WJ-MSCs treated with excessive cortisol displayed similar features as WJ-MSCs from IUGR individuals, when undergoing the chondrogenic differentiation in vitro; (iii) The WJ-MSCs from IUGR individuals presented a poor capacity for chondrogenic differentiation and subsequent increased susceptibility to an osteoarthritis-like phenotype, due to the decreased H3K9ac and expression levels of *TGFβRI* induced by excessive cortisol though GR/HDAC4. Collectively, we proposed that the excessive maternal cortisol induced decreased H3K9ac and expression levels of *TGFβRI* through GR/HDAC4 in utero, which contributed to the poor chondrogenic differentiation of WJ-MSCs from IUGR individuals and subsequently increased susceptibility to an osteoarthritis-like phenotype.

### The decreased H3K9ac level of *TGFβRI* could be an early-warning biomarker for evaluating fetal cartilage development and subsequent susceptibility to osteoarthritis

It has been suggested that the changes of DNA methylation in the liver nuclear factor 4α gene promoter region in the blood stem cells from IUGR umbilical cord exert an essential role in the early onset of diabetes [68]. Early exposure to the unfavorable maternal diets resulted in an altered methylation profile, and transcriptional dysregulation of genes, which were also detectable after birth [69]. Moreover, the human umbilical cord is a tissue that can also be subjected to unfavorable factors during the prenatal period. It has been indicated that the methylation status of the retinoid X receptor alpha promoter in umbilical cord tissue might be utilized to identify individual vulnerability to the later obesity and metabolic diseases [70]. Many studies have explored the early life and childhood risk factors related to osteoarthritis and selected some markers of joint health. Cartilage defects, bone marrow lesions, and meniscal pathology (early markers of joint abnormalities) are known to occur before the clinical symptom and radiographic changes of osteoarthritis, which could be used to predict the development and progression of osteoarthritis in later life [71]. It was also reported that tibial cartilage volume could be a marker of knee joint health in young adults [72, 73]. Besides the cartilage volume, age, and mechanical stress (imbalance in the movement and physical force transmission through the joint), screening epigenetic imprinting as the stable biomarkers from the existing fetal tissues like umbilical cord might be a promising strategy to predict the development of cartilage and the susceptibility to adult osteoarthritis.

For early-life events, epigenetic modification changes might act as one kind of more stable and reliable molecular markers than the expression of target genes [30]. Meanwhile, human umbilical cord is more accessible than other samples from fetuses and could also preserve the epigenetic modification change [70]. Thus, the epigenetic modification changes from the umbilical cord might be the ideal markers for early-life events. In the present study, the decreased H3K9ac level of *TGFβRI* induced by excess cortisol was proved to participate in the poor chondrogenic differentiation of WJ-MSCs from IUGR individuals. Meanwhile, the H3K9ac level of *TGFβRI* and its expression were decreased dramatically both in utero and at the postnatal stage in rat IUGR offspring, which was more susceptible to osteoarthritis in adulthood. Moreover, the H3K9ac level and mRNA expression of *TGFβRI* were all reduced in the umbilical cord from human IUGR newborns. Such findings suggested that the H3K9ac level of *TGFβRI* and its mRNA expression in the WJ-MSCs might be a predictor for cartilage dysplasia and susceptibility to osteoarthritis in the IUGR offspring. Therefore, based on these findings, we recommend the H3K9ac level of *TGFβRI* in human umbilical cord as a potential biomarker for cartilage dysplasia and osteoarthritis susceptibility in the IUGR offspring, although more further evidence is needed. Except for our current study, the early-warning biomarker of fetal-originated adult osteoarthritis has never been reported by far.

## Conclusions

Based on the multipotent differentiation of human WJ-MSCs and the “two-hit” theory in our previous studies, an innovative, two-step cell culture model was established in vitro for investigating fetal-originated adult osteoarthritis. We provided the first evidence that human WJ-MSCs from IUGR newborn exhibited poor capacity of chondrogenic differentiation and the subsequently differentiated chondrocytes presented an increased susceptibility to the osteoarthritis-like phenotype induced by IL-1β, which was attributed to the decreased H3K9ac level and mRNA expression of *TGFβRI* induced by excessive cortisol through GR/HDAC4. Furthermore, we verified that the H3K9ac level of *TGFβRI* could be an early-warning biomarker for predicting cartilage dysplasia and susceptibility to the fetal-originated adult osteoarthritis.

## Supplementary Information

**Additional file 1: Fig. S1.** Characterization of human Wharton’s jelly-derived mesenchymal stem cells (WJ-MSCs). A: The morphology of WJ-MSCs was photographed under a phase-contrast microscope. B-F: Flow cytometric analysis of hematopoietic markers (CD34 and CD45) and the expression of mesenchymal stem cell markers (CD73, CD90 and CD105). **Fig. S2.** MTS analysis of cell viability on 0 and 21th day after chondrogenic differentiation. A: cell viability in control and IUGR groups. *n* = 8. B: cell viability in 300, 600 and 1200 nM cortisol groups. n = 8. Data are mean ± S.E.M. **Fig. S3.** Serum cortisol levels of umbilical cord blood from IUGR and normal individuals by enzyme-linked immunosorbent assay. Control group: *n* = 15, IUGR group: *n* = 14. IUGR, intrauterine growth retardation. Data are the mean ± S.E.M. ^**^P < 0.01 vs control.

## Data Availability

The datasets used and/or analyzed during the current study are available from the corresponding author on reasonable request.

## Abbreviations

IUGR: Intrauterine growth retardation
WJ-MSCs: Wharton’s jelly-derived mesenchymal stem cells
ELISA: Enzyme-linked immunosorbent assay
COL2A1: α1 chain of type II collagen
ACAN: Aggrecan
TGFβRI: Transforming growth factor β receptor I
MMP3: Matrix metalloproteinase 3
ADAMTS5: A disintegrin and metalloprotease with thromospondinmotifs
HDAC: Histone deacetylation
GAPDH: Glyceraldehyde phosphate dehydrogenase
ChIP: Chromatin immunoprecipitation
H3K9ac: Histone 3 lysine 9 acetylation
SPF: Specific pathogen free
GD: Gestational day
PXE: Prenatal xenobiotics exposure
PW: Postnatal week
