# Supplementary material for: H3K9ac of TGFβRI in human umbilical cord: a potential biomarker for evaluating cartilage differentiation and susceptibility to osteoarthritis via a two-step strategy
Source: Stem Cell Res Ther. 2021 Mar 4;12:163. doi: 10.1186/s13287-021-02234-8 (PMC7934528; doi:10.1186/s13287-021-02234-8)
Supplement: Supplementary file 1 — Additional file 1: Fig. S1. Characterization of human Wharton’s jelly-derived mesenchymal stem cells (WJ-MSCs). A: The morphology of WJ-MSCs was photographed under a phase-contrast microscope. B-F: Flow cytometric analysis of hematopoietic markers (CD34 and CD45) and the expression of mesenchymal stem cell markers (CD73, CD90 and CD105). Fig. S2. MTS analysis of cell viability on 0 and 21th day after chondrogenic differentiation. A: cell viability in control and IUGR groups. n = 8. B: cell viability in 300, 600 and 1200 nM cortisol groups. n = 8. Data are mean ± S.E.M. Fig. S3. Serum cortisol levels of umbilical cord blood from IUGR and normal individuals by enzyme-linked immunosorbent assay. Control group: n = 15, IUGR group: n = 14. IUGR, intrauterine growth retardation. Data are the mean ± S.E.M. **P < 0.01 vs control. [file 13287_2021_2234_MOESM1_ESM.docx]

**Supplemental figures and legends:**


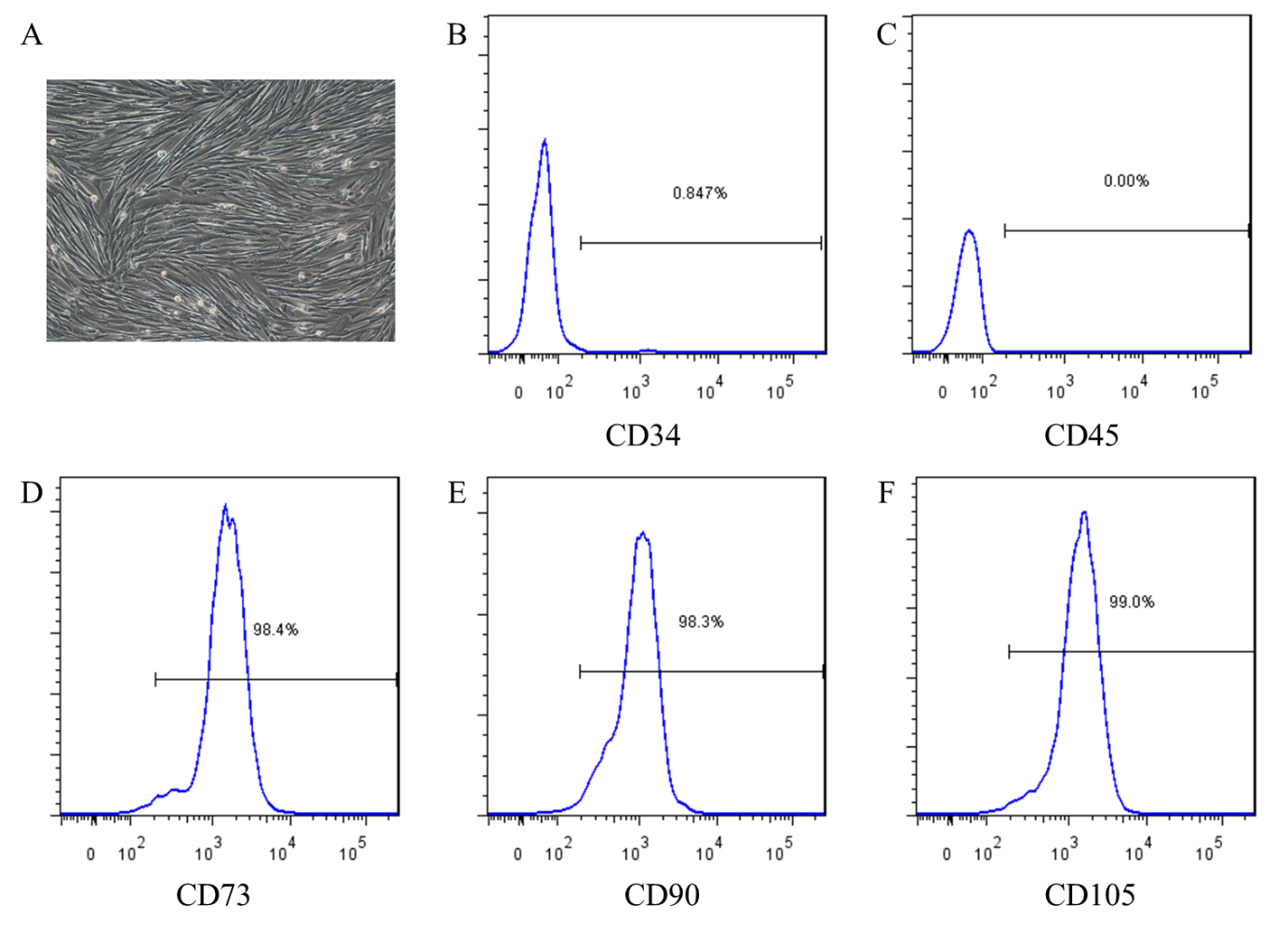


**Fig S1. Characterization of human Wharton's jelly-derived mesenchymal stem cells (WJ-MSCs).** A: The morphology of WJ-MSCs was photographed under a phase-contrast microscope. B-F: Flow cytometric analysis of hematopoietic markers (CD34 and CD45) and the expression of mesenchymal stem cell markers (CD73, CD90 and CD105).


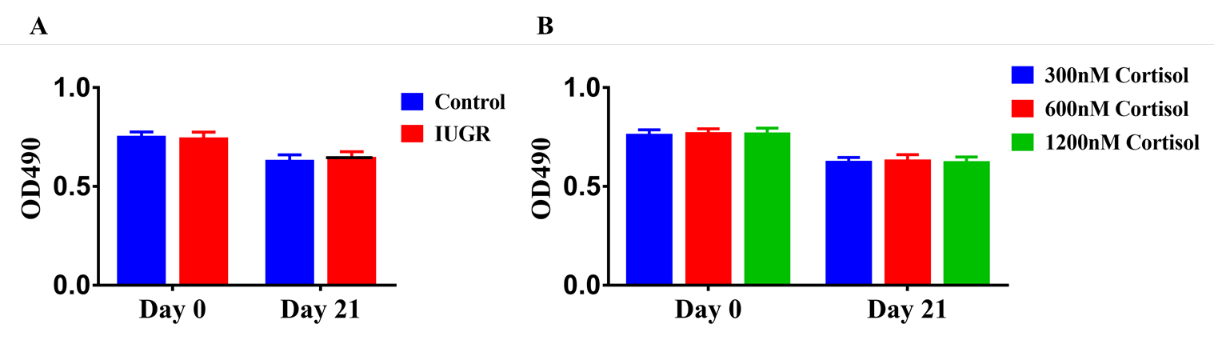


**Fig S2. MTS analysis of cell viability on 0 and 21^th^ day after chondrogenic differentiation.** A: cell viability in control and IUGR groups. n=8. B: cell viability in 300, 600 and 1200 nM cortisol groups. n=8. Data are mean ± S.E.M.


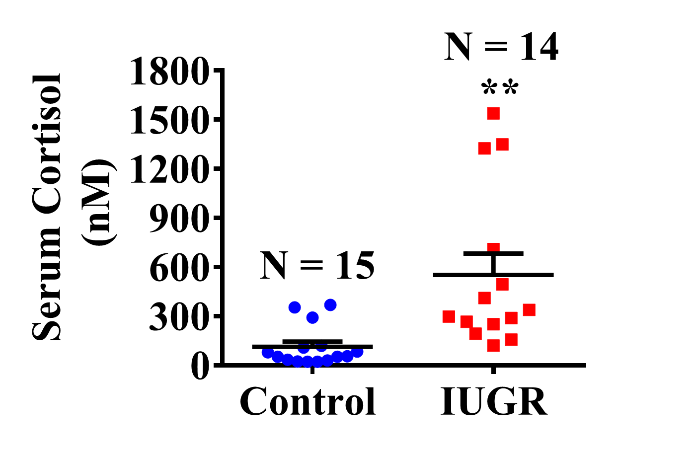


**Fig S3. Serum cortisol levels of umbilical cord blood from IUGR and normal individuals by enzyme-linked immunosorbent assay.** Control group: n=15, IUGR group: n=14. IUGR, intrauterine growth retardation. Data are the mean±S.E.M. ^**^*P<*0.01 *vs* control.
